# The City Initiative for Newborn Health

**DOI:** 10.1371/journal.pmed.0030339

**Published:** 2006-08-08

**Authors:** Armida Fernandez, David Osrin

## Abstract

Fernandez and Osrin describe the first steps taken to revitalize the vast public health system of Mumbai through the active participation of personnel from within the system.

## The Problem

### The challenge of newborn health in India

In India, 26 million babies are born every year, and 1.2 million die in the first four weeks of life (Figure 1)—a figure that accounts for a quarter of global neonatal deaths. India thus faces the biggest newborn health challenge of any country in the world [1]. Survival beyond the newborn period does not mean that the future is assured: about 30% of infants have low birth weight, and over 45% of children between six months and three years old are malnourished [2]. The health of newborn infants affects their subsequent survival [3], growth [4], and cognitive development [5]. It also has profound effects on human capacity, and social and economic development.

**Figure 1:**
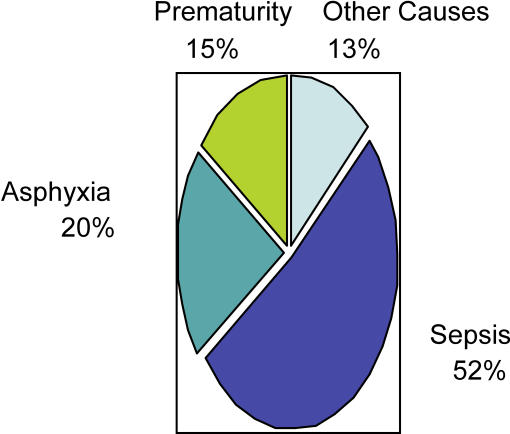
Causes of Neonatal Mortality in India (Adapted from [1])

### The challenge of urbanisation

Rural–urban migration has led to steady urban population growth and a corresponding growth in urban slums (Figure 2). In 1998, India's urban poor outnumbered the rural poor for the first time [6]. For a host of reasons—including problems with water, housing, sanitation, and physical space—the residents of urban slums are affected disproportionately by ill health [7]. In vulnerable situations, children are often the most vulnerable. The prevalence of low birth weight (less than 2,500 g) may be as high as 56% in slum communities [8], compared with a rural level of 38% [9], and an all-India prevalence of 33% [10]. Low-birth-weight babies are more prone to infections and, irrespective of the primary cause of death, account for more than two-thirds of neonatal deaths [1].

**Figure 2:**
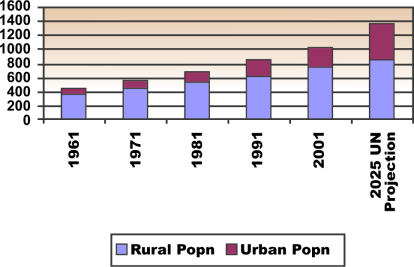
Magnitude of Urban Poverty Worldwide (Data sources: [14,15]) The unit for the y axis is millions. Popn, population; UN, United Nations.

### Mumbai's public health infrastructure

With more than 16.4 million citizens, Mumbai is the most populous city in India. An increase of 50% is predicted between 1996 and 2015. The Municipal Corporation of Greater Mumbai is the primary body responsible for civic administration, covering about 12 million people across five administrative zones and 24 municipal wards. It is also responsible for the city's vast public health infrastructure. This comprises three tertiary hospitals (which are also medical colleges), 13 peripheral hospitals with maternity wards, 25 maternity hospitals, 167 health posts (primary health centres situated in slum areas), and 150 dispensaries. Private health facilities are available for those who can afford them, but the urban poor rely on the public health system, which provides services at nominal rates.

While the public health infrastructure is impressive, there are weaknesses in its provision for mothers and infants, and the special needs of newborn babies are not adequately recognized or addressed. Several interrelated factors are responsible for this weakness. Tertiary hospitals tend to be overburdened as sources of routine prenatal and delivery care; maternity homes—specifically oriented to the management of routine deliveries—are underused; there is limited or no provision of prenatal and postnatal care at health posts; intersectoral linkages are weak and patterns of referral between institutions have not yet been systematised; there is a lack of standardisation of clinical and administrative protocols, particularly in terms of coherence across a range of health-care institutions; care-provider efficiency and morale are low; and the coverage of home-based care and home-visit systems for the vulnerable newborn period is poor.

### Maternity care uptake

Information on maternity practices and care seeking is available from a cross-sectional study undertaken in collaboration with Monitoring and Research Systems. The study interviewed women in Dharavi, familiar to many as one of Mumbai's oldest slum areas, which has seen successive waves of development and community action. Of the respondents, 56% had been married by the age of 18; 63% had three or more children; 35% of female slum residents were illiterate; and most (91%) did not work outside their cramped homes. Registration of pregnancy was high (95%), but late, and only 18% of women had been visited by a health worker during pregnancy. In cases of illness, 52% of women had consulted private practitioners and 34% government services. In addition, 35% of women reported eating less than normal during pregnancy, and 60% reported no change in the amount of rest they took. Although 91% of women had delivered in a health facility—where attendance by health-care professionals was relatively swift and appropriate examination and advice had usually been given—a third had arrived less than an hour before delivery. About one-third of infants were recorded as having low birth weight, and breastfeeding was almost universal but not exclusive.

## The Program

The City Initiative for Newborn Health is an action-research program that aims to improve the survival and health of mothers and newborn infants in slum communities in Mumbai. It has arisen from a collaboration between partner institutions representing four sectors: nongovernmental, governmental, university, and private. The Society for Nutrition Education and Health Action is a nongovernmental organisation, which has a mandate to work with slum communities to improve maternal and newborn health. The Municipal Corporation of Greater Mumbai is the public-sector provider of health services. The International Perinatal Care Unit is an academic research unit based at University College London, United Kingdom, collaborating in applied research to improve maternal and newborn health outcomes. The Social Initiatives Group of ICICI Bank is a development and research unit within one of India's largest banks.

### Core beliefs and components

The initiative's partners believe that a vast and seemingly impersonal public health system can be encouraged to change and become responsive to the critical needs of mothers and newborn infants, that women from the most vulnerable sections of the urban poor can be empowered to seek and demand health care for themselves and their children, and that genuine change requires a sustained and participatory process of inquiry, reflection, and action.

These core beliefs inform the program's two arenas: “demand side” and “supply side”. The first component aims to work with the public health system to achieve continuous quality improvement in maternal and neonatal services at health posts, maternity homes, and hospitals. The second component aims to work with community members in slum areas to improve maternal and newborn-care practices and care seeking. The initiative's pioneering effort, therefore, is to simultaneously affect providers and users, working at scale so that the models it develops will have clear potential for rollout. The first phase (2004–2007) will cover a third of Mumbai's entire public health system.

### The model

One of the innovative aspects of the project is its selection of a methodology that focuses on initiating and sustaining change. The activities within each component involve participation by community members and health-care providers, and it is through them that the need for change is articulated. This requires a conviction that the participants themselves, their workplaces, and their social milieus have the potential to change, and that they can be directly involved in planning and implementation.

The initiative has based its approaches at all levels on a process of “appreciative inquiry”, the philosophy of which is to look at the existing strengths of individuals in an organisation or community and their potential as a nexus of positive attributes for creating a desired future. Appreciative inquiry is used increasingly in public health initiatives [11,12]. Each program component will centre on the formation and activities of groups. These groups may be institutional or community-based, and have a range of structures that allows for cross-disciplinary discussion and involvement of key stakeholders. Appreciative inquiry usually moves through four stages: the group discovers periods of excellence and achievement and thus identifies its strengths; the group dreams an ideal organisation or community, based on existing strengths and grounded in reality; the group designs new structures and processes to realise this dream; and the group takes responsibility for delivering the dream through their implementation. The process is participatory and democratic, and encourages ownership.

### Continuous quality improvement at health posts, maternity homes, and hospitals

One of the most challenging tasks is to create a sustainable and coherent referral system. In keeping with the program ethos, action groups have been formed for each level of institution: maternity homes, peripheral hospitals, and tertiary hospitals. Each group is made up of personnel from different cadres, including doctors, nurses, administrators, and paramedical and domestic staff. Over the first 18 months, eight action groups have used appreciative inquiry to envision and implement a number of tasks. The groups oversaw a baseline survey of health posts, maternity homes, and municipal hospitals, in which—for the first time—comprehensive and accurate data were collected on human resources, equipment, drug supply, record systems, and transport facilities.

Two examples of the findings were a comparison of high- and low-performing maternity homes and an analysis of the transfer of clients from one level of facility to a higher level. The cost per bed at a high-performing facility with an occupancy of 103% was IRs165. At a low-performing facility with an occupancy of 18%, the cost was IRs820. The clients' perceptions of the reasons for which they were transferred included clinical indications (44%), lack of staff (36%), and lack of equipment (8%). There were 12% of clients who did not know the reason for transfer. From a provider's perspective, 26% of transfers from peripheral to tertiary hospitals were the result of a lack of anaesthetic cover.

The current aim is standardisation for tiers of facility in a range of areas: clinical and nursing protocols for maternal and neonatal care, facility roles and requirements (human resources, equipment, drugs, and consumables—all classified into one of three categories: vital, essential, and desirable), and administrative services. Action groups have developed protocols for clinical, administrative, and facility upgrading—basing their work on best-practice guidelines and consultation with local experts from all levels of facility and regions of the city.

The process of implementing clinical protocols has begun, and the next step is to strategise for the mobilisation of resources required. Action groups have come up with 86 strategies for intervention, which have been subsequently grouped into shorter- and longer-term options. Work has begun on 35 short-term options, with the aim of generating buy-in through early achievement. Upgrading facilities involves groups in brokering the use of health department, private-sector, and charitable resources. One interesting strategy aims to improve public-sector information technology and connectivity. Another is to mediate training. Master trainers have been identified and key topics prioritised, with an emphasis on skill-based training methods. Obstetric topics include postpartum haemorrhage, monitoring of labour, identification and management of foetal distress, and management of pregnancy-induced hypertension. Neonatal topics include routine care, resuscitation, management of jaundice, convulsions, hypoglycaemia, and sepsis.

Communication between personnel at all levels has been supported by a series of wider workshops—the idea being that the referral system will be operationalised by personnel who recognise themselves as members of a collective, united by a common purpose. The referral system aims to distribute the case load so that complicated cases will receive prompt and timely care at an appropriate level and that the current pressure on tertiary facilities is eased, while underused peripheral hospitals will achieve higher access.

### Community mobilisation

Simultaneously with health service activities, a process of community mobilisation is being activated. The initiative will work with community groups to improve maternal and newborn health in vulnerable urban slums. The aim is to effect change in care practices and health-care-seeking behaviour for women in the prenatal, delivery, and postpartum periods, and for newborn infants, with subsequent effects on newborn and maternal illness and mortality. The initiative has conducted studies to identify particularly vulnerable urban slum areas, within which program facilitators will help to convene community groups to explore health issues. The role of the facilitator is to activate and strengthen groups, support them in identifying and prioritising maternal and newborn problems, help to identify possible solutions, and support the planning, implementation, and monitoring of the solution strategies in the community.

## Evaluation

The implementation of public health programmes is a point at which the need for evidence meets the need to justify expenditure. Since the City Initiative is an exploratory endeavour, evaluation is crucial. The first phase will run for four years and will evaluate its activities with a range of methods. Continuous quality-improvement activities at maternity homes and peripheral and tertiary hospitals will be evaluated through process documentation, comparison of the situation in one urban zone before and after intervention, and comparison of the situation in one zone with other municipal zones. Simultaneously, the health post and community mobilisation interventions will be evaluated through a cluster-randomised controlled trial. Ethically, the City Initiative for Newborn Health is a partnership with the Municipal Corporation of Greater Mumbai, and corporation staff is involved in all interventions. Benefits to trial control areas will be improvements in services at all levels of the public health-care system.

## Applicability in Other Settings

Mumbai is both typical and special. In some ways it is the archetypal megacity, with extensive slums, overcrowding, and a public-service system constantly trying to cope with a growing burden. In other ways, it is unusual. Firstly, it is affluent: constant immigration is driven by its dynamism and possibility of betterment. If you can make it there, you'll make it anywhere. Secondly, it has a particularly structured and accountable municipal corporation, which has relatively good health services for an urban Indian setting.

Can the City Initiative model be applied in other towns and cities? The model itself is a real-world one. It engages directly with both public-sector workers and community members in the context of existing pressures like low morale, salary difficulties, absenteeism, and ossified organisational cultures. For this reason, as a mode of entry and as leverage for improvement, it may be a good option for any setting. It is crucial to balance the supply and demand sides of the health equation. On the supply side, the group-driven appreciative model seems to be (a) generic and (b) popular with participants. On the demand side, there is evidence that community groups can improve mother and child health [13], but most of the work to date has been rural rather than urban. It remains to be seen—and this is why evaluation is so important—whether participatory models can succeed in a range of settings. The critical first steps have been taken in an exciting but challenging attempt to improve urban health in India, and we need to sustain the impetus and learn from the outcomes.
